# Global prevalence of congenital heart disease in school-age children: a meta-analysis and systematic review

**DOI:** 10.1186/s12872-020-01781-x

**Published:** 2020-11-19

**Authors:** Yingjuan Liu, Sen Chen, Liesl Zühlke, Sonya V. Babu-Narayan, Graeme C. Black, Mun-kit Choy, Ningxiu Li, Bernard D. Keavney

**Affiliations:** 1 Division of Cardiovascular Sciences, University of Manchester, AV Hill Building, Manchester, M13 9PT UK; 2 Department of Social Medicine, West China School of Public Health, Sichuan University, Chengdu, China; 3 Division of Paediatric Cardiology, Department of Paediatrics, Red Cross War Memorial Children’s Hospital, University of Cape Town, Cape Town, South Africa; 4 Division of Cardiology, Department of Medicine, Groote Schuur Hospital, University of Cape Town, Cape Town, South Africa; 5 National Heart and Lung Institute, Imperial College London, Royal Brompton and Harefield NHS Trust, London, UK; 6 Manchester Academic Health Science Centre, Manchester University NHS Foundation Trust, Manchester, UK; 7 Division of Evolution and Genomic Sciences, University of Manchester, Manchester, UK

**Keywords:** Congenital heart disease, Prevalence, School children, Meta-analysis, National income

## Abstract

**Background:**

Congenital heart disease (CHD) is the commonest birth defect. Studies estimating the prevalence of CHD in school-age children could therefore contribute to quantifying unmet health needs for diagnosis and treatment, particularly in lower-income countries. Data at school age are considerably sparser, and individual studies have generally been of small size. We conducted a literature-based meta-analysis to investigate global trends over a 40-year period.

**Methods and results:**

Studies reporting on CHD prevalence in school-age children (4–18 years old) from 1970 to 2017 were identified from PubMed, EMBASE, Web of Science and Google Scholar. According to the inclusion criteria, 42 studies including 2,638,475 children, reporting the prevalence of unrepaired CHDs (both pre-school diagnoses and first-time school-age diagnoses), and nine studies including 395,571 children, specifically reporting the prevalence of CHD first diagnosed at school ages, were included. Data were combined using random-effects models. The prevalence of unrepaired CHD in school children during the entire period of study was 3.809 (95% confidence intervals 3.075–4.621)/1000. A lower proportion of male than female school children had unrepaired CHD (OR = 0.84 [95% CI 0.74–0.95]; *p* = 0.001). Between 1970–1974 and 1995–1999, there was no significant change in the prevalence of unrepaired CHD at school age; subsequently there was an approximately 2.5-fold increase from 1.985 (95% CI 1.074–3.173)/1000 in 1995–1999 to 4.832 (95% CI 3.425–6.480)/1000 in 2010–2014, (*p* = 0.009). Among all CHD conditions, atrial septal defects and ventricular septal defects chiefly accounted for this increasing trend. The summarised prevalence (1970–2017) of CHD diagnoses first made in childhood was 1.384 (0.955, 1.891)/1000; during this time there was a fall from 2.050 [1.362, 2.877]/1000 pre-1995 to 0.848 [0.626, 1.104]/1000 in 1995–2014 (*p* = 0.04).

**Conclusions:**

Globally, these data show an increased prevalence of CHD (mainly mild CHD conditions) recognised at birth/infancy or early childhood, but remaining unrepaired at school-age. In parallel there has been a decrease of first-time CHD diagnoses in school-age children. These together imply a favourable shift of CHD recognition time to earlier in the life course. Despite this, substantial inequalities between higher and lower income countries remain. Increased healthcare resources for people born with CHD, particularly in poorer countries, are required.

## Background

Congenital heart disease (CHD) is the commonest birth defect. The advent of modern cardiological and cardiac surgical care has transformed the prognosis for even critical types of congenital heart disease in high-income countries. Approximately 85% of children born with CHD now survive to adulthood in high income countries; over 1 million adults are now thought to be living with CHD in the USA [1].

Previous literature-based meta-analyses [2, 3] have shown global increases in the birth prevalence of CHD, but consistently low estimates of birth prevalence in poorer countries. These findings have chiefly been attributed to increased availability of diagnostic technology in middle-income countries, coupled with ongoing missed diagnoses of CHD in early life in severely resource-limited contexts. “Missing cases” at birth, however, indicate only part of the potential shortfall in healthcare availability for children with CHD. In this regard, global data regarding trends in access to definitive treatment for children diagnosed in infancy are relatively sparse. Individual studies of school-age children have reported CHD prevalence varying widely, from 0.5/1000 to 18/1000 [4, 5]. By contrast with studies of live births with CHD, which have been conducted using large birth registries, the sample sizes of studies in school children have generally been relatively modest, which limits the inference possible from any individual investigation.

Multiple studies have found that in poorer countries, delayed diagnosis of CHD is a major issue [6, 7]. Late presentation with avoidable severe complications (for example, development of the Eisenmenger phenomenon in patients with large septal defects) is known to contribute significantly to adverse outcomes in these settings [8]. To explore the global magnitude of the shortfall in treatment of known CHD cases, we estimated the prevalence of unrepaired CHD at school age and described the associated time trends. Additionally, we reasoned that global falls in new CHD diagnoses at school age, occurring in lockstep with previously reported global increases in birth prevalence, would support the notion that higher reported birth prevalences are indeed a result of greater availability of diagnostic technology, as opposed to changes in population susceptibility. We synthesised data from the literature to address these questions, exploring heterogeneity over time, by national income, and by geographic location.

## Methods

The review was conducted and reported according to the PRISMA statement [9]. The full review protocol is presented in Additional file 1.

### Search strategy

We considered all papers published between January 1970 and June 2017 in 4 databases, PubMed, EMBASE, Web of Science and Google Scholar. The main search items used were: “Congenital heart disease” or “Congenital heart defect” or “Heart abnormality” or “Heart malformation” and “Prevalence” or “Incidence” or “Frequency” or “Epidemiology”. We excluded studies performed before 1970 as echocardiography for CHD was not widely applied at that time [10, 11].

### Data review

All three stages of review (of the titles, abstracts and full texts) were done by two people (YL and SC). Any discrepancy was resolved by discussion. All unique papers (peer-reviewed publications with English abstracts) reporting on CHD prevalence of school-age children (4–18 years old) were included. Studies focusing on higher-risk groups, such as children with Trisomy 21, or twins, were excluded. The details of the inclusion/exclusion criteria are shown in Additional file 1: Table S1. Of note, there was no overlap in data between the present study and our previous meta-analysis of the birth prevalence of CHD [3]. Three papers [12–14] which had measured both birth and childhood prevalence, were included in both meta-analyses. Data from these three studies were partitioned depending on age group classification as birth/early infancy, or school-age, and contributed to only the previous report [3], or the present study respectively.

**Fig. 1:**
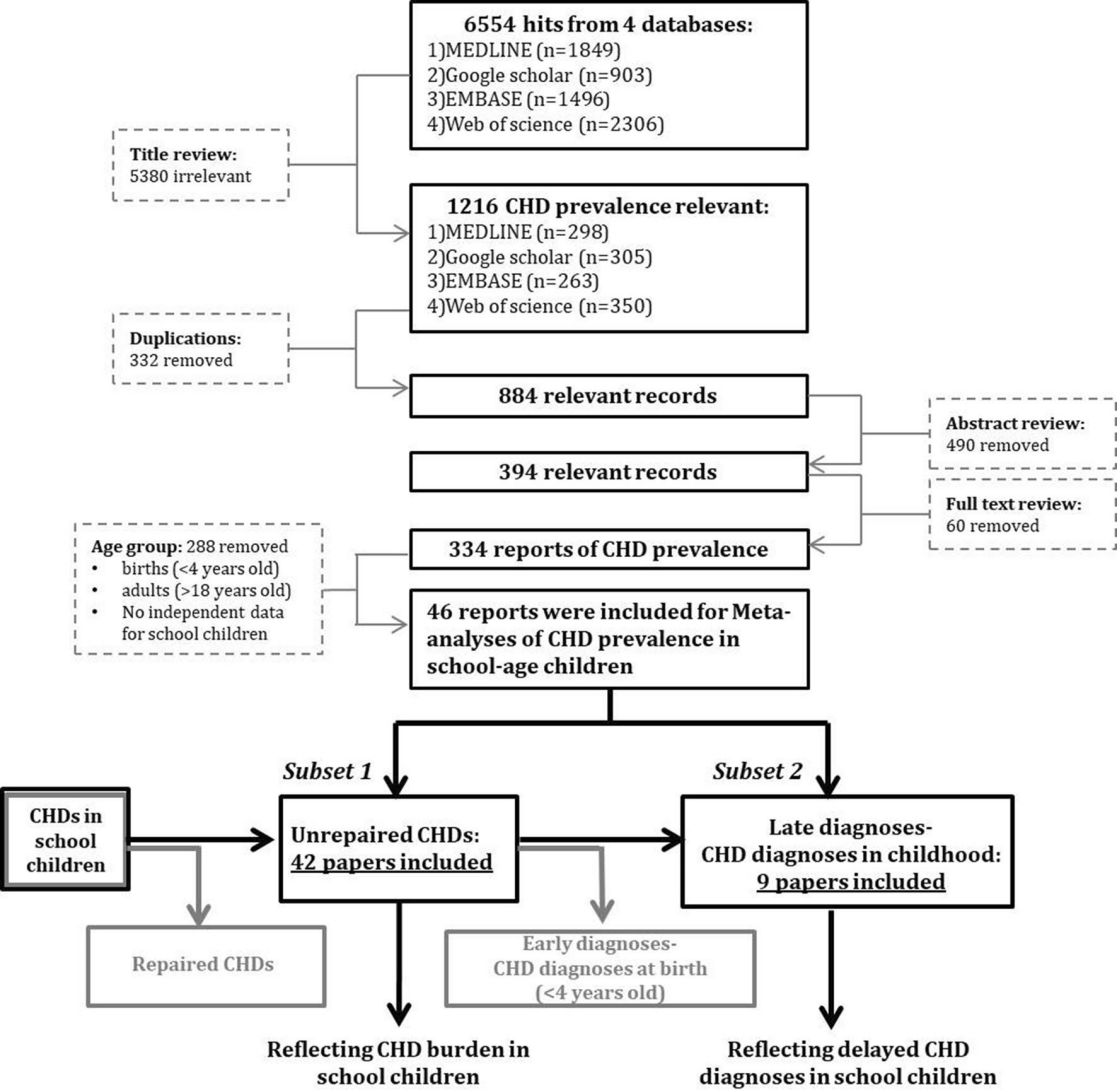
The PRISMA flow chart and schema of Congenital heart diseases (CHDs) prevalence processed in meta-analyses. Two different questions were addressed respectively by two subsets of CHD prevalence data: unrepaired CHDs (CHDs repaired with surgical care or interventions were excluded) regardless of time of diagnosis, and CHD diagnoses in childhood (delayed CHD diagnoses at school age). Repaired CHDs and early diagnoses (both in grey) were not analysed in late diagnoses of this review

Two meta-analyses were performed (Fig. 1). The meta-analysis of unrepaired CHDs yielded the trend in prevalence of unrepaired CHD in school children. The meta-analysis of CHDs newly diagnosed owing to the participant’s enrollment in one of the included studies—of necessity, a subset of unrepaired CHDs—yielded the trend in delayed diagnoses of CHDs in school-age populations.

### Data extraction and quality assessment

Data were extracted independently by two reviewers (YL and SC). Any discrepancy between two reviewers was resolved by discussion. Information about the prevalence of CHD overall, and of 27 CHD subtypes, was screened and recorded. The quality of included studies was assessed with the Newcastle–Ottawa Scale (NOS, maximum 10 stars for cross-sectional studies), which is recommended by the Cochrane Collaboration and widely applied for quality assessment on observational studies [15–17]. Raw data and NOS scores are available in Additional file 2.

For time trend analysis of changes in CHD prevalence, studies were subdivided into nine groups of 5 years according to their investigation time. None of the included studies contained data collected after 2014. For time-trend analysis of subtypes, severe CHD was defined based on Hoffman’s classifications [18–20]. (Additional file 1: Table S2). Atrial Septal Defects (ASD) and Ventricular Septal Defects (VSD) were combined as “Septal defects”.

The Gross National Income (GNI) per capita (Atlas method) for countries were collected from the database of The World Bank [21]. Countries were classified as low income (≤ $1025 pa), lower middle income ($1026–$4035 pa), upper middle income ($4036–$12,475 pa) and high income (≥ $12,476 pa) [22].

### Data analysis

Data were analyzed with R Meta package and STATA 14. Random-effect models for summarizing CHD prevalence were applied since our a priori expectation was of a highly heterogeneous dataset. Pre-specified subgroup analyses estimating the association between prevalence of CHD and geographic regions and income levels were performed using Chi-square tests. In subgroup analyses, the *p* values were adjusted using the Bonferroni method. In time trend analyses, the “metareg” command was used on study-level data. The significance level was pre-specified as 0.05 (two-tailed). The data in the text is presented as mean (95% CI).

## Results

A total of 46 studies were included in the review, adhering to the processes shown in the PRISMA flow chart (Fig. 1). The included studies were of cross-sectional design with a mean NOS score of 6.96 ± 1.15 (mean ± SD, range 5–9).

**Fig. 2:**
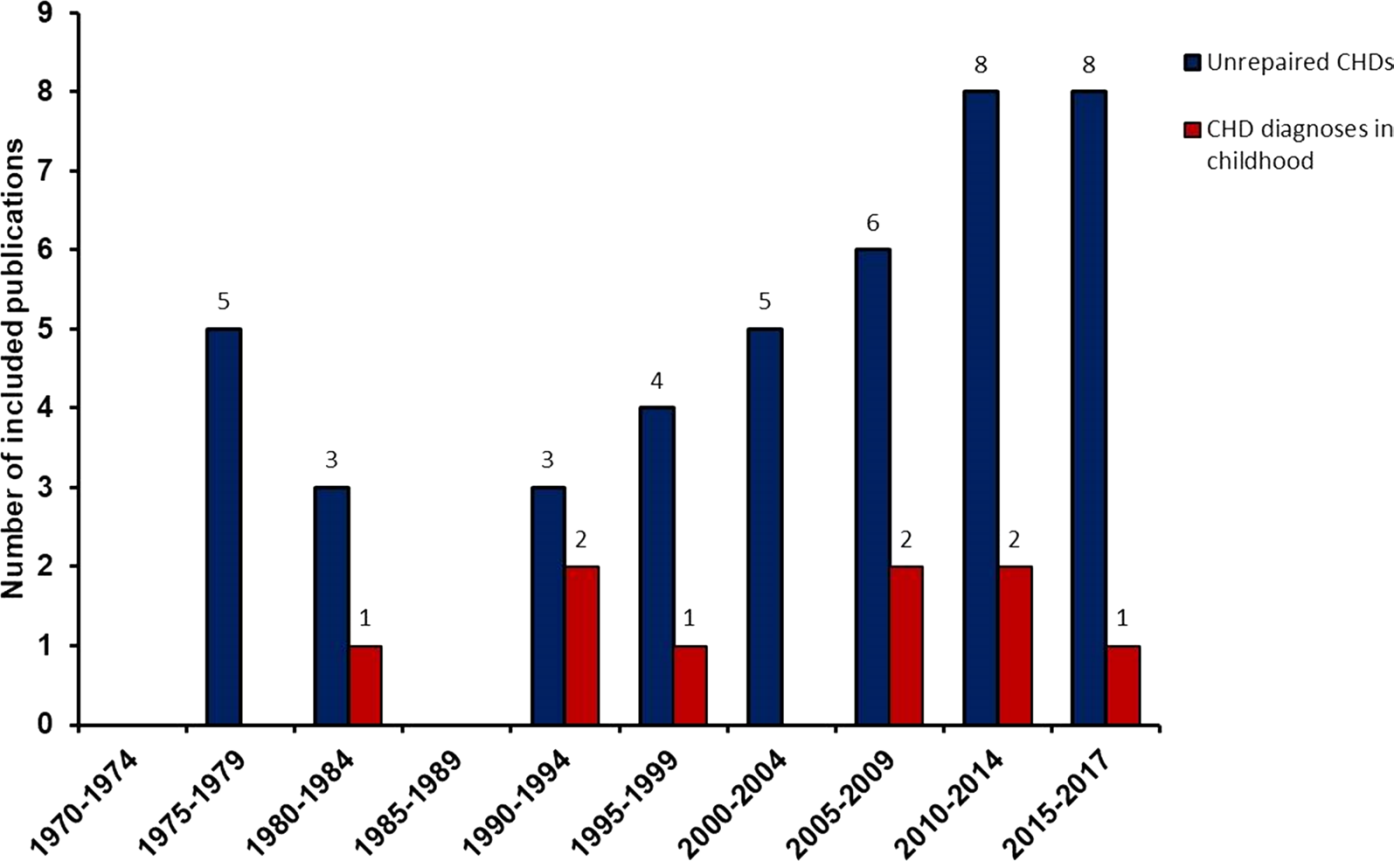
The number of included studies published in 5-year bins 1970–2017. The time bins were grouped according to the publication time of studies

The publication years of the included studies are shown in Fig. 2. Of note, no eligible studies were published in the years 1970–1974; however, three included studies published 1975–1977 collected data from 1970 onwards and contributed to the 1970–1974 data bin. Sample sizes varied widely; the majority of studies were conducted in small populations, with only seven involving hundreds of thousands of children. In studies where the diagnostic methodology was reported (91.3%), 78.6% overall had used echocardiography; after 1995, 93.5% of studies used echocardiography. In total, 42 studies including 2,638,475 children, among whom a total of 5406 were unrepaired CHD cases, reported on the overall prevalence of unrepaired CHDs (both pre-school diagnoses and first-time diagnoses at school ages). Data relating to CHD diagnoses newly made at school age were sparser—only nine studies including 395,571 children, among whom 376 were new CHD cases diagnosed at school age, were included. Four of those studies reporting on new CHD diagnoses at school age (242 cases) came from a single country (Thailand) at different time periods. Five papers yielded information on both unrepaired (203 cases) and school-age diagnosed CHD (134 cases), so the data were partitioned and contributed to both analyses.

**Fig. 3:**
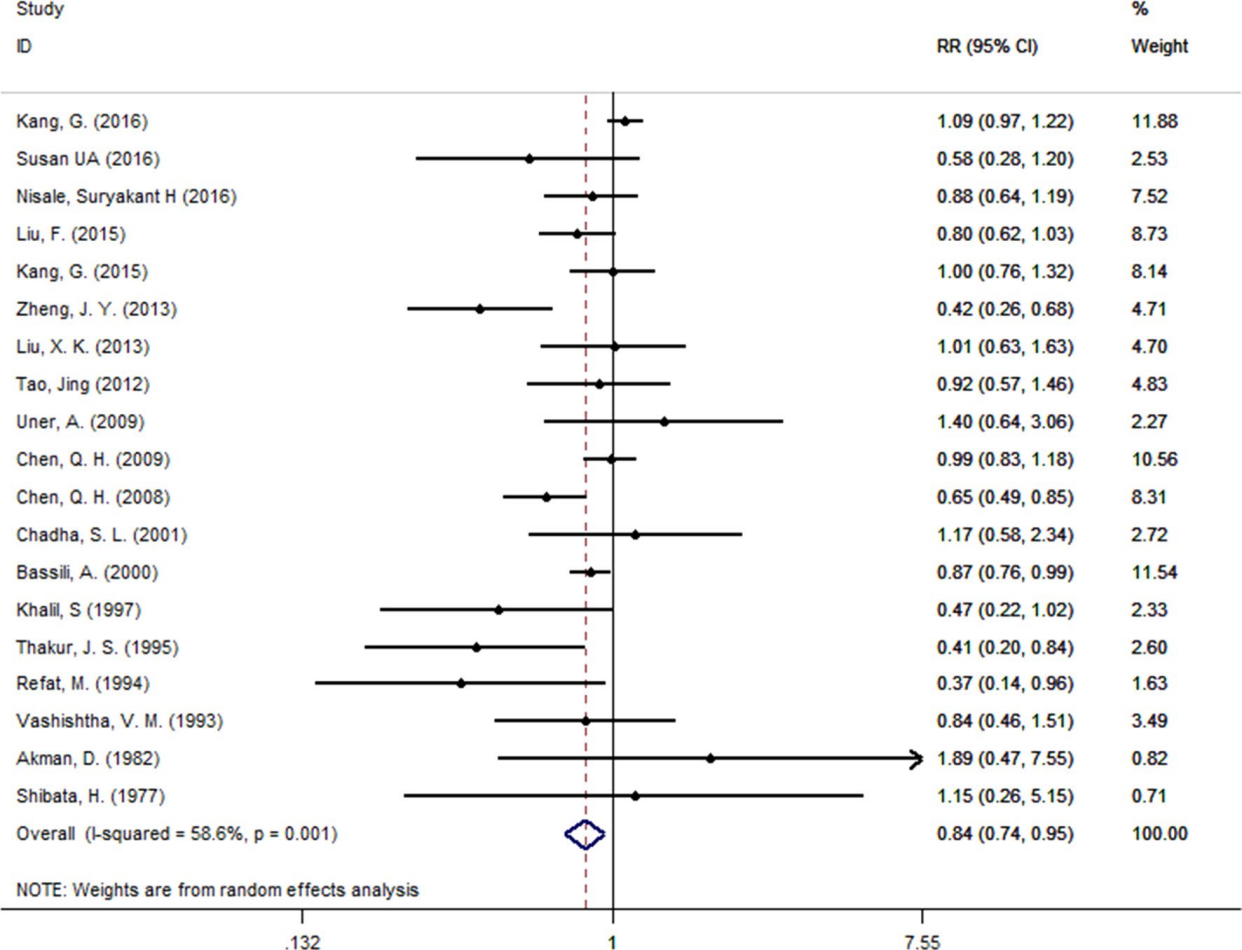
The male to female ratio for unrepaired CHD prevalence in school children. Values < 1 reflect the lower proportion of males

**Fig. 4:**
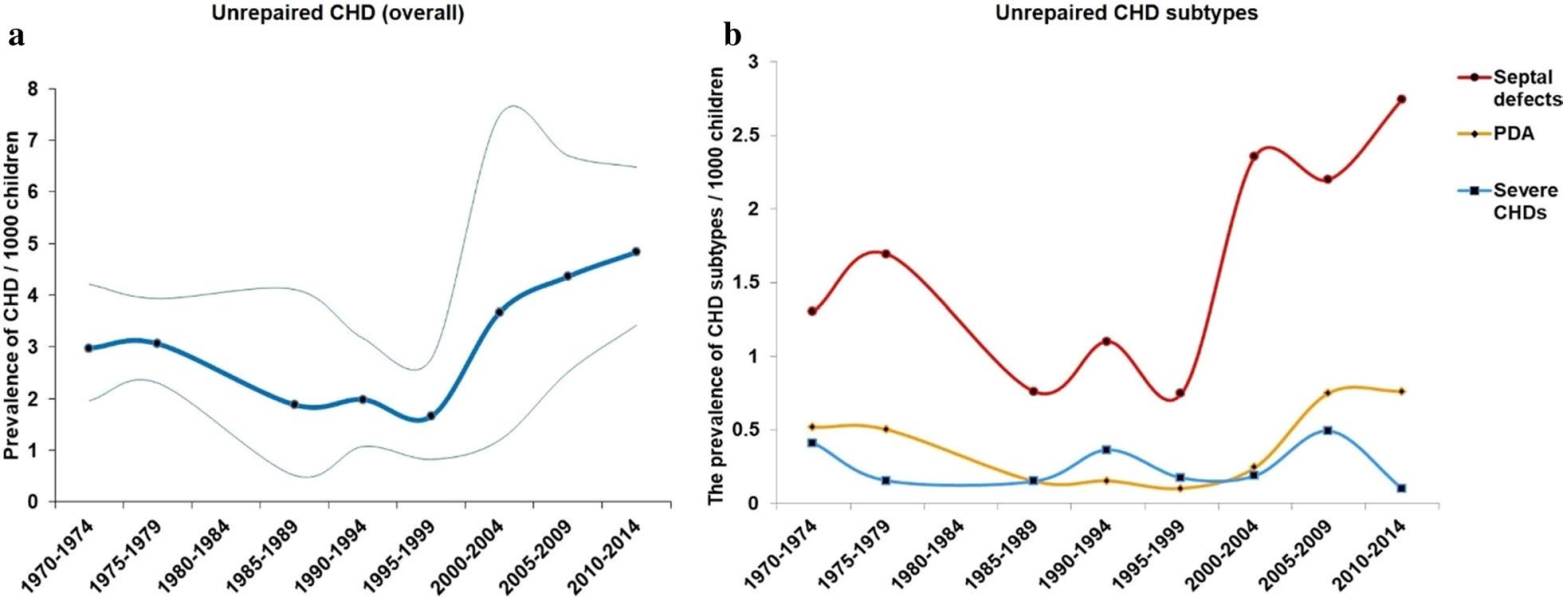
Changes in the prevalence of unrepaired CHD and CHD subtypes in school age children during 1970–2014. **a** The prevalence of unrepaired CHD overall during the studied period. Thick dark blue line is the average value of prevalence, while the thin lines are 95% CIs. **b** The prevalence of unrepaired Septal defects (VSD and ASD), PDA and severe CHDs of the entire period. Prevalence of Septal defects, PDA and severe CHDs were represented in red, orange and light blue lines with dots, respectively. The time bins are defined by investigation time reported by studies

The prevalence of unrepaired CHDs throughout the study period was 3.809 (3.075, 4.621)/1000 with, as anticipated, high heterogeneity (*I*^2^ = 98.8%) among studies. The prevalence of unrepaired CHD in males (3.381 [2.792, 3.971]/1000) was lower than in females (4.083 [3.440, 4.726]/1000); RR = 0.84 (0.74, 0.95), *p* = 0.001 (Fig. 3). Among 42 studies reporting on unrepaired CHDs, 28 studies were conducted in Asia, where the prevalence of unrepaired CHDs was 3.531 [2.554, 4.666]/1000, and nine in Africa, where the prevalence was 4.350 [2.385, 6.898]/1000. Within Asia and Africa, the prevalence of unrepaired CHDs in low/middle income countries (3.841 [3.016, 4.765]/1000) was higher than in high income countries (1.779 [1.409, 2.193]/1000); *p* < 0.001. In trend analyses, a significant rise in prevalence of unrepaired CHD was observed since 1995, from 1.657 (0.824, 2.777)/1000 in 1995–1999 to 4.832 (3.425, 6.480) in 2010–2014; *p* = 0.009 (Fig. 4a).

The breakdown of prevalence by 27 subtypes of CHD is shown in Table 1. As expected, the commonest types of unrepaired CHD were VSD (30.3% of overall prevalence) and ASD (24.5% of overall prevalence), followed by patent ductus arteriosus (PDA, 13.9%). The prevalence of unrepaired septal defects (VSD and ASD) and PDA rose (*p* value for both < 0.05) after 1995, while the prevalence of severe unrepaired CHD lesions was unchanged (Fig. 4b).

**Table 1:** The prevalence and percentages of 27 CHD subtypes

CHD subtypes	Unrepaired CHDs	
	Prevalence of CHD subtypes/1000 (95% CIs)	Percentage of CHD subtypes, % (95% CIs)
Ventricular septal defect	0.997 (0.711–1.331)	30.342 (25.651–35.249)
Atrial septal defect	0.902 (0.659–1.183)	24.456 (19.803–29.431)
Patent ductus arteriosus	0.516 (0.349–0.715)	13.896 (10.348–17.871)
Pulmonary stenosis	0.230 (0.151–0.325)	7.229 (4.827–10.071)
Aortic stenosis	0.243 (0.132–0.388)	8.548 (5.313–12.465)
Mitral insufficiency	0.385 (0.174–0.679)	9.182 (3.631–16.926)
Tetralogy of fallot	0.167 (0.112–0.233)	5.613 (3.918–7.590)
Coronary artery aneurysm	0.001 (0.000–0.006)	0.113 (0.003–0.631)
Dextrocardia	0.111 (0.014–0.300)	3.555 (0.867–7.967)
Tricuspid atresia or stenosis	0.131 (0.039–0.275)	5.567 (1.34–12.433)
Aortic valve insufficiency	0.069 (0.017–0.157)	5.471 (1.38–12.046)
Ebstein anomaly	0.011 (0.003–0.026)	0.960 (0.393–1.774)
Congenital heart block	0.164 (0.000–1.545)	1.389 (0.003–5.223)
Pulmonary arteriovenous aneurysm	0.011 (0.005–0.018)	0.913 (0.434–1.568)
Pulmonary atresia	–	–
Mitral stenosis	0.049 (0.005–0.139)	4.710 (0.141–15.166)
Endocardial cushion defect	0.033 (0.022–0.045)	2.803 (1.672–4.215)
Transposition of the great arteries	0.025 (0.010–0.048)	2.113 (1.320–3.088)
Coarctation of the aorta	0.087 (0.012–0.229)	1.750 (0.562–3.581)
Hypoplastic left heart syndrome	0.083 (0.002–0.464)	1.429 (0.036–7.704)
Single ventricle	0.009 (0.004–0.018)	0.908 (0.393–1.781)
Truncus arteriosus	0.007 (0.003–0.015)	0.681 (0.250–1.476)
Cor triatriatum	0.012 (0.000–0.069)	0.505 (0.012–2.782)
Double outlet right ventricle		
Partial anomalous pulmonary venous return	–	–
Total anomalous pulmonary venous return	–	–
Interrupted aortic arch	–	–

**Fig. 5:**
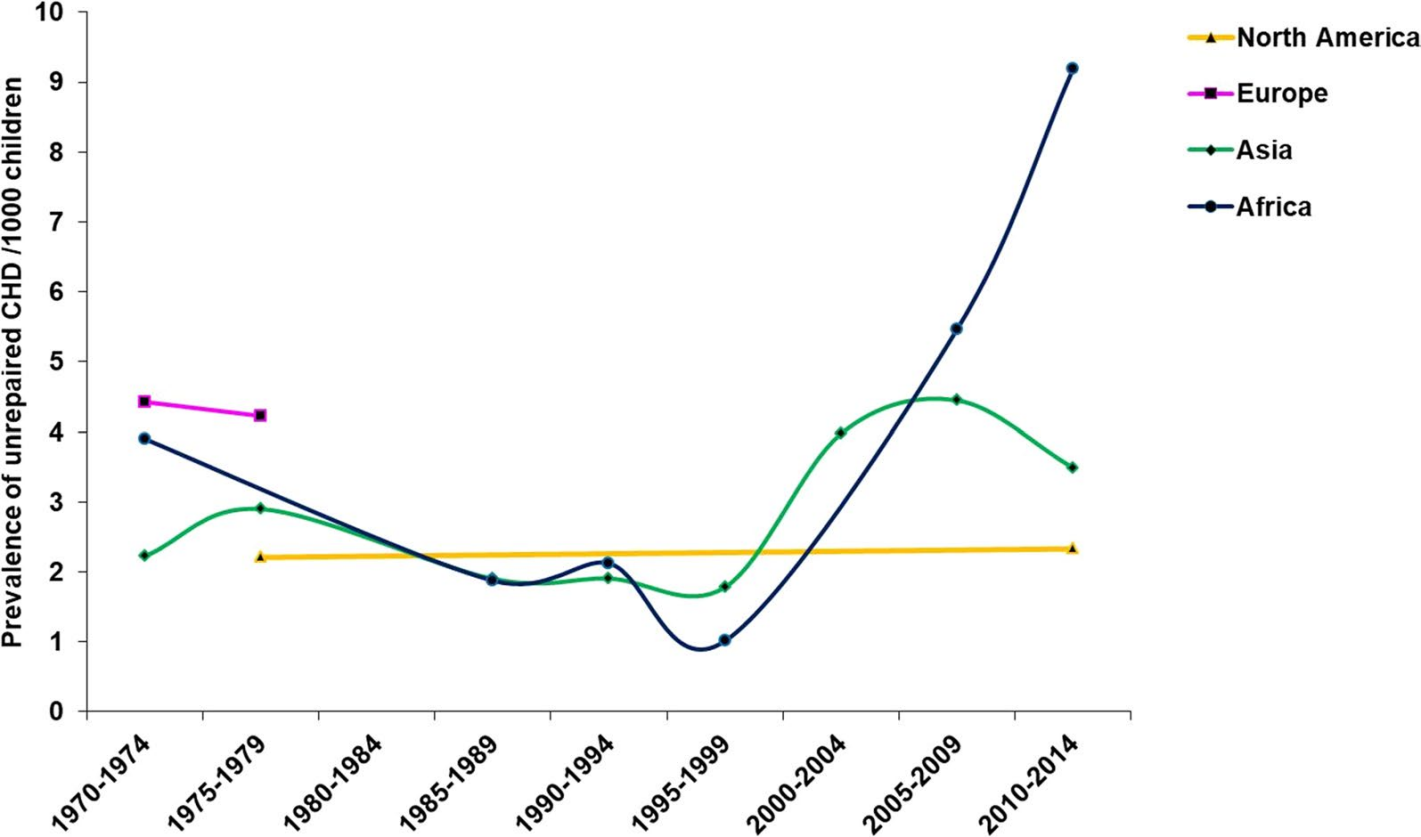
The changes in unrepaired CHD prevalence among school children in 4 geographic regions. The number of studies for each region was: Africa 10 (920,691 children), Asia 27 (1,538,294 children), Europe 2 (55,985 children), North America 2 (122,482 children). One study conducted in South America comprising just 1023 children, is not shown in the figure.

Analyses by geographical region showed a highly significant increase in the reported prevalence of unrepaired CHD in Asia (*p* = 0.034) and Africa (*p* = 0.029), between 1995 and the present (Fig. 5). By the most recent period, 2010–2014, Africa had the highest prevalence of unrepaired CHD compared to both Asia and North America in 2010–2014, and compared to Europe in 1975–1979, the last period data was available.

**Fig. 6:**
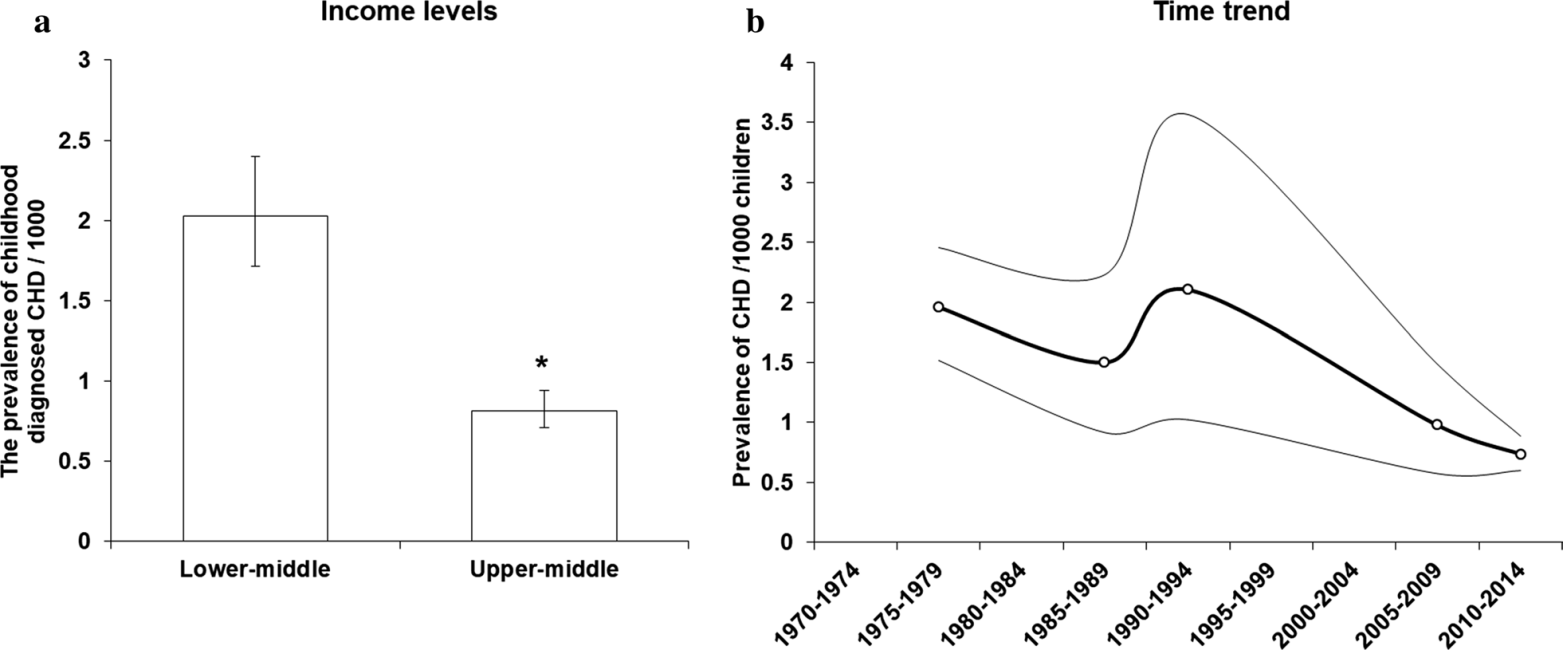
The prevalence of CHD diagnoses in childhood for countries of different income levels and geographic regions. **a** prevalence difference between income levels across the study period. Data in **a** are presented as Mean + SE. *P < 0.05. **b** The prevalence of CHDs diagnosed in childhood from 1970–2014. Thin lines represent 95% CIs. *P* value for trend analysis is 0.040

All nine studies reporting data on CHD diagnoses in childhood were from Asia and Africa and involved lower-middle and upper-middle income countries only. The overall prevalence of CHDs diagnosed in childhood in 1970–2014 was 1.384 (0.955, 1.891)/1000, suggesting these represented 36.3% [31.4%, 41.2%] of unrepaired CHDs in the period. Analyses grouping studies by income status showed a higher prevalence of childhood diagnosed CHD in lower-middle than upper-middle income countries (Fig. 6a). In contrast to the increasing trend of total unrepaired CHD, a fall in prevalence of CHD diagnoses in childhood (*p* = 0.04), from 2.050 [1.362, 2.877]/1000 pre-1995 to 0.848 [0.626, 1.104]/1000 in 1995–2014, was shown in these nine studies, albeit with a small total number of CHD cases (Fig. 6b).

## Discussion

We present the first literature-based meta-analysis of the global prevalence of unrepaired CHD in school children. Our findings provide information on diagnostic trends over a 30-year period, and illustrate aspects of the residual unmet need for CHD care, particularly in developing countries. Strikingly, studies included in this meta-analysis suggest that the prevalence of recognised but unrepaired CHD among school-age children in the world has more than doubled since 1995. This is mainly accounted for by increased diagnosis of septal defects (VSD and ASD) and PDA in Asia and particularly in Africa. Many of the newly diagnosed children with these milder forms of CHD (e.g. VSD spontaneous closures) do not necessarily need surgeries or interventions, but specialist practitioners providing the lifelong follow-up these patients may require are insufficient in countries of the Global South [23]. Increased early recognition of CHDs in childhood avoids first time diagnoses in adulthood with complications that could have been avoided. Such presentations, including infective endocarditis, a cardiovascular crisis in pregnancy, or the development of pulmonary hypertension, are not uncommon in low- and middle-income countries (LMICs).

The increasing trend of unrepaired CHD prevalence in school-age children corresponds with our previous results in a global meta-analyses of birth prevalence; both suggests that substantially increased numbers of diagnoses in infancy and childhood are being made in LMICs [3]. Improvements in socio-economic status in many countries since the 1970s, accompanied by increased access to public health provision, are clearly important contributory factors to improved diagnostic rates. More specifically, enhanced quality and greater availability of echocardiography seem likely to be major drivers for the increased diagnoses of mild CHD conditions [24, 25]. In settings of limited resources, portable echocardiography may in the future offer significant further diagnostic advance in CHD, since it can be deployed in local clinics in rural settings, and instruments capable of producing diagnostic images in CHD are available at low cost [26, 27]. Regarding CHD specifically, Sulafa and Tajudeen’s evaluation, for example, supports a high agreement (91.7%) of diagnoses made using either handheld or standard echocardiography [28]. Further research will be required to attribute findings proportionately among the factors contributing to increased diagnostic rates, which is beyond the scope of the present study.

We found significantly fewer males with unrepaired CHD among school children (RR 0.84; *p* = 0.001). The incidence of CHD among males and females at birth is similar; previous studies have shown a higher CHD mortality rate among males in infancy than females which could account for our result [4, 29]. However, we cannot entirely rule out differential access to surgery between male and female infants as a contributory cause. Further work will be required to identify the causes of this observation.

Data contributing to our analyses are mainly from countries in Asia or Africa. Within the limited range of countries represented, we found significantly higher prevalence of unrepaired CHD in lower income countries than higher income countries. The same was true in the studies focused specifically on CHDs diagnosed for the first time at school age, although data on this latter question were sparser. These differences of CHD prevalence between countries in different income groups highlight the ongoing lack of availability of diagnostic resource, and accessibility of surgical or medical services for CHD, in lower income countries. While most developed countries benefit from foetal echocardiography with high detection sensitivity of 60–80% [30] in the past few decades, these diagnostic techniques, devices and services are still rarely affordable or available to poorer countries. Lower health education levels in poorer countries constitute an additional obstacle for improving early CHD detection and optimising management of CHD patients requiring long-term care [31].

Corresponding with the increasing prevalence of overall CHDs which the present study has shown at school-age, and other studies have demonstrated at birth [3], the limited data available regarding CHD diagnosed for the first time at school ages shows this has decreased since the 1970s, by approximately 75%. This difference suggest a shift of CHD recognition time from school age to pre-school age or neonatally. Mild lesions, like ASDs, do not typically produce symptoms in early life; they are easily missed in screening at early infancy, and commonly recognised later in childhood or young adults [32]. However, the advantages of recent echocardiography has enhanced the sensitivity of CHD detection and diagnostic quality of even small lesions [33]. Although our dataset could not explore the likely consequences of late diagnoses of CHD for patients, these data suggested the improved technology has brought forward the recognition time of CHDs, especially mild CHDs. Avoidable and life-threatening complications may result from the delayed diagnosis of lesions that are straightforward to repair—such as large VSDs which if diagnosed late may present with the Eisenmenger phenomenon [34]. Thus, public health initiatives aimed at early diagnosis using simple and widely applicable technology, such as portable pulse oximetry [35], may have particular benefits in developing countries.

This study has limitations. Perhaps the most significant is the sparse data available on CHD diagnoses first made in childhood among school children. There are insufficient data (mainly from Thailand) to draw inferences beyond the statistically significant fall over time of CHD diagnoses made as a result of participating in prevalence surveys at school age. A second limitation is that we cannot translate the prevalence of unrepaired CHDs in our dataset directly into estimates of unmet need for surgical or interventional cardiological care, since some CHDs (small ASDs, VSDs, and PDAs) may not require correction or may resolve spontaneously. Nevertheless, best practice would require these children to have a periodic follow-up with specialist paediatric, and later with adult congenital cardiologists; access to these services is known to be severely limited in poorer countries. We excluded post-operative cases, as the principal aim of the work was to comment upon unrepaired lesions. Of course, patients who have undergone palliation or incomplete correction for complex lesions also contribute significantly to the burden of CHD morbidity in children and young adults; further studies will be necessary to assess the degree to which the needs of these patients are met in different socio-economic conditions, and at different ages.

## Conclusions

The reported prevalence of unrepaired CHDs in low-income countries is increasing in recent decades, especially in Africa. The similar increasing trends of recognised CHDs in both school-age children and live births, and corresponding reductions in first-time CHD diagnoses in school-age children, indicate a beneficial shift in CHD recognition time from school-age to earlier stages. Additional resources are needed to be allocated to the care of the increasing number of children recognised to be living with unrepaired CHDs.

## Supplementary information

**Additional file 1.** Review protocol and supplementary tables for inclusion and exclusion criteria and classification of severe CHDs.

**Additional file 2.** List of included studies and extracted original data for meta-analyses.

## Data Availability

The datasets supporting the conclusions of this article are included within the article as Additional file 2.

## Abbreviations

CHD: Congenital heart defect
NOS: Newcastle–Ottawa Scale
GNI: Gross National Income
VSD: Ventricular septal defect
ASD: Atrial septal defect
PDA: Patent ductus arteriosus
LMICs: Low- and middle-income countries
