# Supplementary material for: Global prevalence of congenital heart disease in school-age children: a meta-analysis and systematic review
Source: BMC Cardiovasc Disord. 2020 Nov 19;20:488. doi: 10.1186/s12872-020-01781-x (PMC7678306; doi:10.1186/s12872-020-01781-x)
Supplement: Supplementary file 1 — Additional file 1. Review protocol and supplementary tables for inclusion and exclusion criteria and classification of severe CHDs. [file 12872_2020_1781_MOESM1_ESM.docx]

**Review protocol:**

**Title: Global prevalence of Congenital Heart Disease in school-age children: a meta-analysis and systematic review**

**Review questions:**

1. What is the global prevalence of congenital heart diseases (CHDs) in school-age children?

2. Where have the studies on CHD prevalence of school-age children been conducted?

3. When is the time of CHD diagnosis for school-age children?

4. Is there a gender difference in CHD prevalence between male and female school-age children?

5. What is the influence of social factors (geographic regions and incomes) on CHD prevalence of school-age populations?

**Searches:**

The following electronic databases will be systematically searched :

PubMed, EMBASE, Web of Science and Google Scholar.

The search strategy will combine the following key words:

Disease: “Congenital heart disease” or “Congenital heart defect” or “Heart abnormality” or “Heart malformation”

Topic: “Prevalence” or “Incidence” or “Frequency” or “Epidemiology”.

Time: “Publication time: Jan. 1970-Jun.2017”

Species: not “animal”

Design: “cross sectional” or “cohort” or “case control”

Type: not “review”

There is no language restriction

Example of search in the database PubMed:

*((((((((((((analysis, cross sectional[MeSH Terms]) OR analysis, cohort[MeSH Terms]) OR case control studies[MeSH Terms])) AND ((((((((((((prevalence[MeSH Terms]) OR incidence[MeSH Terms]) OR frequency[MeSH Terms]) OR epidemiology[MeSH Terms])) AND ((((((((congenital heart defect[MeSH Terms]) OR congenital heart disease[Text Word]) OR congenital heart defect[Text Word] OR abnormality, heart[MeSH Terms]) OR heart abnormality[Text Word]) OR heart abnormalities[Text Word]) OR heart malformation[Text Word]))) AND ("1970/01/01"[Date - Publication] : "2017/06/30"[Date - Publication]))) NOT review[Text Word]) NOT animal[Text Word])))))))))*

**Type of studies to include:**

All relevant peer-reviewed observational studies reporting on CHD prevalence in school-age children (4-18 years old) will be reviewed. All designs of original studies (except reviews), including cohort, cross-sectional and case-control studies, to be considered.

**Population being studied:**

School age children with an age range of 4-18 years old will be included.

**Exposure:**

The review aims to evaluate the general population prevalence of CHD in school children. Therefore, publications dealing specifically with populations exposed to known CHD risk factors, including teratogens, maternal diabetes, prenatal maternal infections, multiple conceptions, consanguineous marriages, or dealing with known syndromic conditions predisposing to CHD (for example Down’s syndrome) are excluded.

**Context:**

This review summaries on the prevalence of overall CHDs and 27 subtypes recorded in ICD-10. This review includes the information of study sample sizes, number of CHD cases, number of CHD subtypes, diagnosis time and method used, study locations, and investigation time. The distribution of CHD in different regions and its changes along with time are described.

**Data extraction:**

***Study selection.*** Relevant studies to be reviewed by YL and SC according to the following inclusion/exclusion criteria. The reviews are processed in three stages: titles, abstracts and full texts. Review of titles is conducted before the combination of records from 4 databases, while review on abstracts and full texts is performed after combination of databases and removing of duplications. Any discrepancy is resolved by discussion and referral back to the original context of papers.

***Inclusion criteria and exclusion criteria are listed in Table S1.***

***Data coding.*** The extraction/coding form was developed to collect all data about study characteristic (Author, publication time, investigation time, locations, study designs, sampling and diagnosing methods), prevalence data (sample sizes, genders, CHD cases and 27 CHD subtypes). Data was recorded by YL and SC.

**Risk of bias (quality) assessment**

All the included papers are evaluated on their reporting quality with [The Newcastle-Ottawa Scale (NOS)](http://www.ohri.ca/programs/clinical_epidemiology/oxford.asp) according to the study design of papers.

**Data synthesis strategy**

Based on the general guideline of narrative synthesis (Rogers, et al. 2009), a preliminary synthesis was developed first to explore the relationships between studies. The preliminary synthesis indicated high heterogeneity within studies. Therefore, random effect model was applied for meta-analyses.

**Subset/subgroup analyses**

***Subsets of interest.*** Unrepaired CHDs were defined as all CHDs reported that had not been subject to operations or percutaneous interventions. New diagnoses of CHD, made as a result of participation in one of the included studies, were recorded separately.

***Subgroup analyses.*** In analysing the time trends of CHD prevalence, the studies were allocated to 9 year-groups, according to their investigation years. To identify the potential influence of social factors on the prevalence of CHDs, the studies were classified by geographic regions and income levels. The classification of regions and income levels were based on the Internet World Stats ( https://www.internetworldstats.com/list1.htm ) and The World Bank ( https://datahelpdesk.worldbank.org/knowledgebase/articles/906519-world-bank-country-and-lending-groups).

Table S 1 The inclusion and exclusion criteria for review

| **Inclusion criteria** | **Exclusion criteria** |
| --- | --- |
| - The subjects were human beings. | - Studies on animals. |
| - Studies was conducted with a focus on prevalence of CHD. | - Studies did report on overall CHD prevalence or focus on severe or other CHD subgroups only. |
| - Papers should report the total number of CHD cases and sample sizes, or these numbers could be calculated from the reported data. |  |
| - Studies were original papers and published on peer reviewed journals with English abstracts. | - Papers of reviews, comments or books. |
|  | - Results was non-original but estimation from other data sources. |
|  | - No English abstracts. |
|  | - Conference abstracts without full text papers. |
| - Sampling from unbiased populations. | - Studies about the prevalence of CHD in special populations , like Down syndrome population, offspring from mothers with Diabetes, Children with other defects, twins, data collected from Intensive cate unit, or other situations with known factors which can changes the risk of CHD. |
| - The investigation time of studies was not earlier than 1970. | - Studies conducted before 1970. |
| - The population were aging of 4-18, which is age range for school children. | - Studies on children or adults with the age < 4 years old and >18 years old, or the data for school children could not be separated from overall data. |

Table S 2 Classification of severe CHDs based on Hoffman's method (2002) and modified according to methods from Alexander (2014) and Raluca (2009)

| Severe CHD | Subtypes |  | ICD 10 Code |
| --- | --- | --- | --- |
| Cyanotic lesions | 1. D-transposition of the great arteries | | Q20.3 |
|  | 2. Tetralogy of Fallot | | Q21.3 |
|  | 3. Right heart lesions  a. Tricuspid atresia  b. Pulmonary atresia  c. Ebstein anomaly | | Q22.4  Q22.0  Q22.5 |
|  | 4. Hypoplastic left heart syndrome  5. Interrupted aortic arch | | Q23.4  Q25.2 |
|  | 6. Single ventricle | | Q20.4 |
|  | 7. Double outlet right ventricle | | Q20.1 |
|  | 8. Truncus arteriosus | | Q20.0 |
|  | 9. Total anomalous pulmonary venous connection | | Q26.2 |
| Acyanotic lesions | Atrioventricular septal defect | | Q21.2 |
